# Supplementary material for: A Novel Pathogenic Variant in MT-CO2 Causes an Isolated Mitochondrial Complex IV Deficiency and Late-Onset Cerebellar Ataxia
Source: J Clin Med. 2019 Jun 4;8(6):789. doi: 10.3390/jcm8060789 (PMC6617079; doi:10.3390/jcm8060789)
Supplement: Supplementary file 1 [file jcm-08-00789-s001.pdf]

Journal of
Clinical Medicine

MDPI

Supplementary Table 1. Summary of previously reported cases with pathogenic variants in the MT-CO2 gene.

Publication	Age of onset	Clinical features	Variant	Heteroplasmy	Maternal inheritance
Rahman et al. 1999	14 years	Myopathy, lactic acidosis	m.7671T>A	Muscle (90%), blood (5%)	Possible de novo (not detectable in mother's blood)
Clark et al. 1999 Proband	5 years	Progressive gait ataxia, cognitive impairment, OA, RP	m.7587T>C	Muscle (91%)	Yes
Mother	47 years	Cerebellar ataxia	m.7587T>C	Muscle (67%)	Yes
Wong et al. 2001	At birth (born prematurely)	Apnoea, bradycardia and severe lactic acidosis. The other twin brother died similarly.	m. 8042delAT	Muscle (20%), cardiac (17%), lung (14%), liver (14%), skin (12%)	Possible maternal based on family pedigree but not detectable in mother's blood
Campos et al. 2001	3 months	Psychomotor delay, failure to thrive, RP, LVH, lactic acidosis	m.7896G>A	Muscle (76%), blood (67%), fibroblast (60%)	Possible de novo (not detectable in blood of mother and sister)
Uusimaa et al. 2002	6 months	Failure to thrive, Alper-like disease, cardiomyopathy, lactic acidosis	m.7706G>A	Muscle (90%), blood (87%), kidney (90%), liver (91%), brain (91%)	Yes; blood in asymptomatic mother (72%) and grandmother (66%)
McFarland et al. 2004	30 years	Recurrent rhabdomyolysis	m.7989T>C	Muscle, not detectable in blood	Possible de novo (not detectable in mother's blood)
Horvath et al. 2005	15 years	Short stature, myopathy, cataract, SNHL, cardiac arrhythmia, lactic acidosis, ataxia and acute encephalopathy	m.7970G>T	Muscle (90%)	Yes; detectable in blood and urine samples of sister and niece (10-15%)
Rossmanith et al. 2005	13 years	MELAS, LVH, myoclonus, basal ganglia calcification	m.7630delT	Muscle (93%), blood (28%)	Possible de novo (not detectable in blood of mother and other siblings)
Zhadanov et al. 2006	24 years	LHON, epilepsy	m.7598G>A	Blood (80%)	Yes; blood in asymptomatic mother (25%)
Vissing et al. 2013	Childhood	Recurrent myoglobinuria, abnormal acylcarnitidine profile (MADD)	m.8156dupG	Muscle (56%); not detectable in blood, urine, buccal and fibroblast	Possible de novo

Kytovuori et al. 2017		Deafness, progressive cognitive decline, epilepsy, psychosis, exercise intolerance, RP, cataract (in early 20s),	m.8156delG	Muscle (95%), buccal (33%), blood (26%)	Possible de novo (not detectable in blood of mother and other siblings)
Roos et al. 2019	14 years	Myopathy, exercise intolerance and abnormal acylcarnitidine profile (MADD)	m.8088delT	Muscle (96%), hair (2%), blood (5%), fibroblast (6%), urine (15%), buccal (12%)	Possible de novo (not detectable in multiple non-invasive tissues of mother)

LHON= Leber hereditary optic neuropathy, LVH= left ventricular hypertrophy, MADD= multiple acyl-CoA-dehydrogenase deficiency, OA= optic atrophy, RP= retinitis pigmentosa, SNHL= sensorineural hearing loss, MELAS= mitochondrial encephalomyopathy, lactic acidosis and stroke like episode.

Journal of
Clinical Medicine

References

1. Rahman S, Taanman JW, Cooper JM, et al. A missense mutation of cytochrome oxidase subunit II causes defective assembly and myopathy. American journal of human genetics. 1999 Oct;65(4):1030-9.
2. Clark KM, Taylor RW, Johnson MA, et al. An mtDNA mutation in the initiation codon of the cytochrome C oxidase subunit II gene results in lower levels of the protein and a mitochondrial encephalomyopathy. American journal of human genetics. 1999 May;64(5):1330-9.
3. Wong LJ, Dai P, Tan D, et al. Severe lactic acidosis caused by a novel frame-shift mutation in mitochondrial-encoded cytochrome c oxidase subunit II. American journal of medical genetics. 2001 Jul 22;102(1):95-9.
4. Campos Y, Garcia-Redondo A, Fernandez-Moreno MA, et al. Early-onset multisystem mitochondrial disorder caused by a nonsense mutation in the mitochondrial DNA cytochrome C oxidase II gene. Annals of neurology. 2001 Sep;50(3):409-13.
5. Uusimaa J, Finnila S, Vainionpaa L, et al. A mutation in mitochondrial DNA-encoded cytochrome c oxidase II gene in a child with Alpers-Huttenlocher-like disease. Pediatrics. 2003;111(3):e262-8.
6. McFarland R, Taylor RW, Chinnery PF, Howell N, Turnbull DM. A novel sporadic mutation in cytochrome c oxidase subunit II as a cause of rhabdomyolysis. Neuromuscular disorders: NMD. 2004 Feb;14(2):162-6.
7. Horvath R, Schoser BG, Muller-Hocker J, Volpel M, Jaksch M, Lochmuller H. Mutations in mtDNA-encoded cytochrome c oxidase subunit genes causing isolated myopathy or severe encephalomyopathy. Neuromuscular disorders: NMD. 2005 Dec;15(12):851-7.
8. Rossmanith W, Freilinger M, Roka J, et al. Isolated cytochrome c oxidase deficiency as a cause of MELAS. BMJ case reports. 2009;2009.
9. Zhadanov SI, Atamanov VV, Zhadanov NI, Schurr TG. De novo COX2 mutation in a LHON family of Caucasian origin: implication for the role of mtDNA polymorphism in human pathology. Journal of human genetics. 2006;51(3):161-70.
10. Vissing CR, Duno M, Olesen JH, et al. Recurrent myoglobinuria and deranged acylcarnitines due to a mutation in the mtDNA MT-CO2 gene. Neurology. 2013 May 14;80(20):1908-10.
11. Kytovuori L, Karppa M, Tuominen H, et al. Case report: a novel frameshift mutation in the mitochondrial cytochrome c oxidase II gene causing mitochondrial disorder. BMC neurology. 2017 May 18;17(1):96.
12. Roos S, Sofou K, Hedberg-Oldfors C, et al. Mitochondrial complex IV deficiency caused by a novel frameshift variant in MT-CO2 associated with myopathy and perturbed acylcarnitine profile. European journal of human genetics: EJHG. 2019 Feb;27(2):331-5.
